# Characterization of the role of Ume6 C-terminal tail in *C. albicans* morphological plasticity

**DOI:** 10.17912/micropub.biology.000827

**Published:** 2023-05-25

**Authors:** Ben Evans, Evan Spell, Douglas Bernstein

**Affiliations:** 1 Biology, Ball State University, Muncie, Indiana, United States

## Abstract

*C. albicans* is an important human fungal pathogen and filamentation is essential for its virulence. Ume6 is a transcription factor critical for filamentation. Ume6 is composed of three domains, a long N terminal domain, Zn-finger domain, and a C-terminal domain. Previously, it was shown that the Zn-finger domain is essential for filamentation, as removal of this domain led to a lack of filamentation. However, the role for the C-terminal domain has not been defined. We find deletion of the C-terminal domain leads to a filamentation defect and the defect is not as severe as removal of the Zn-finger or *ume6* deletion. We mutated a number of residues in the C-terminal domain to try to identify specific residues important for filamentation, but all of our mutants displayed wild type filamentation. Alpha fold predictions suggest the C-terminal domain forms a single alpha helix that is predicted to interact with the Zn-finger domain via hydrogen bond. Our data suggests the C-terminal domain binds the Zn-finger domain and through this interaction is important for filamentation.

**Figure 1. Domain structure, growth, and filamentation of Wild Type C. albicans and UME6 mutants.**
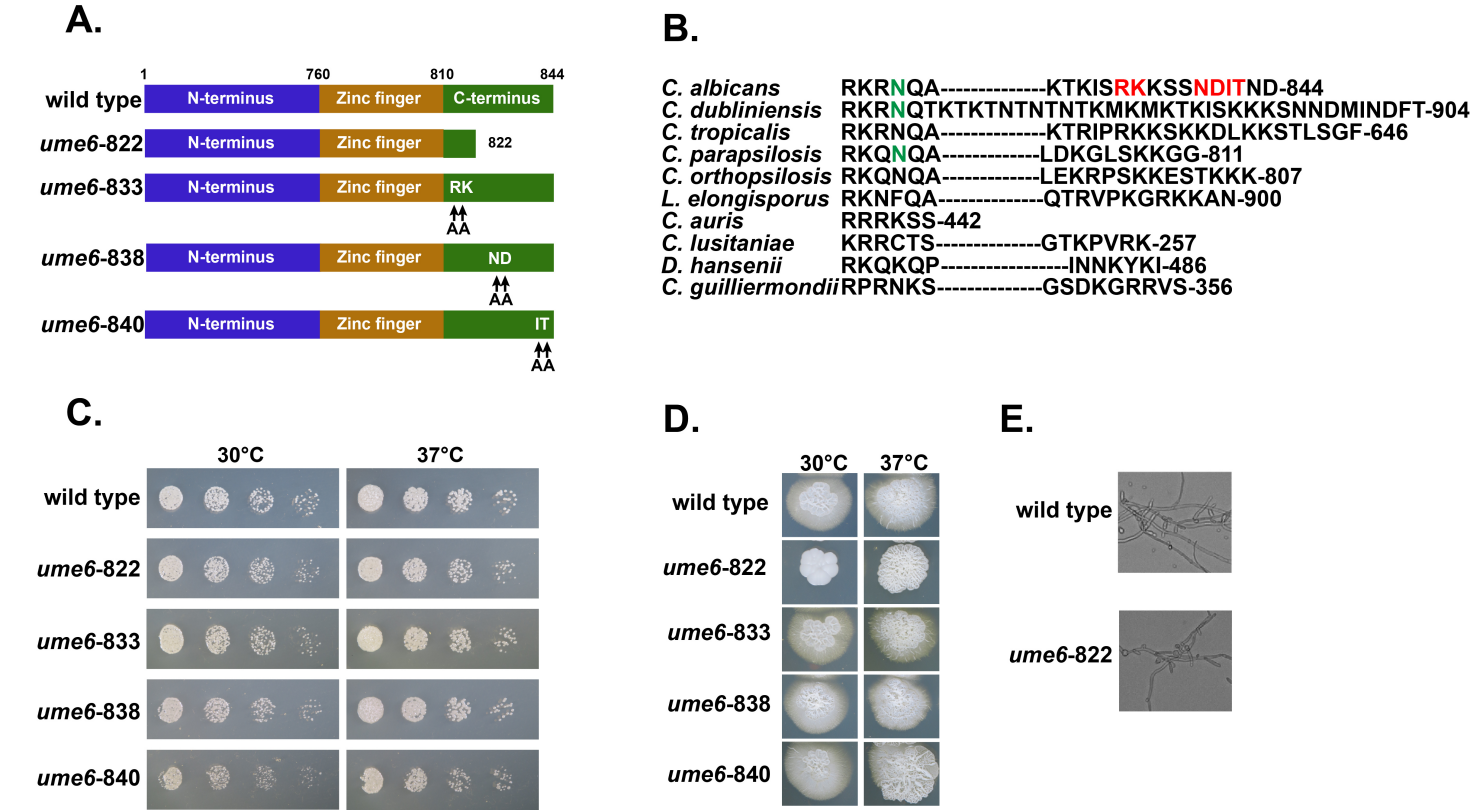
**A.** Domain structure of wild type *C. albicans* Ume6, truncation mutants, and point mutants used in this study. **B.** Alignment of C-terminal tails of CTG clade Ume6 proteins. Residues that have been changed to alanine in the mutants are colored red. The residue that we found to interact with the Zinc-finger domain via hydrogen bond are colored green. **C.** Serial dilution growth assays of wild type, *ume6-822* , *ume6-833* , *ume6-838* , and *ume6-840* . Cultures were grown for 24 hours on YPD at 30 and 37 C°. **D.** Filamentation assays of wild type, *ume6-822* , *ume6-833* , *ume6-838* , and *ume6-840.* Cultures were grown for 9 days on Spider media at 30 and 37 C°. **E.** Wild type and *ume6* -822 grown in Spider media for 24 hours at 37 C°.

## Description

*C. albicans* is the most prevalent human fungal pathogen (Beck-Sague and Jarvis 1993). As a commensal, *C. albicans* asymptomatically colonizes gut mucosal tissue, however, immunosuppression can lead to *C. albicans* becoming an opportunistic pathogen. *C. albicans* can infect a wide range of host tissues including the mouth, genitourinary tract, heart, and kidneys. Infections range in severity from irritating mucosal infections, such as thrush, to candidemia, a life-threatening systemic infection (Pfaller and Diekema 2010) . Invasive candidiasis in immunocompromised individuals has an estimated mortality of 40% (Wey et al. 1988; Viudes et al. 2002) and as such it is critical that we better understand the underlying molecular processes required for virulence.

In response to stress, *C. albicans* , like many human fungal pathogens, can undergo morphological changes and these changes are critical for virulence. One of the most pronounced morphological changes *C. albicans* undergoes is the process of filamentation. During filamentation, *C. albicans* produces long extensions that can invade agar or tissue (Arkowitz and Bassilana 2019) . These filamentous extensions can branch, forming a complex network of filaments, or form buds thus reverting to oval yeast cell morphology. One protein known to be important for activation of filamentation is Ume6 (Banerjee et al. 2008) .

*C. albicans* Ume6 is a transcription factor that is over 800 amino acids in length. It is composed of a 760 amino acid long but poorly conserved N-terminal domain **Figure 1A** (Skrzypek et al. 2017) . C-terminal to this lies a Zinc-finger domain thought to be important for DNA binding **Figure 1A** . The Zinc-finger is critical for function as truncation of this domain leads to loss of *C. albicans'* ability to form filaments. C-terminal to the Zinc-finger domain is a poorly conserved C-terminal domain for which little is known **Figure 1B** . Given its lack of conservation, we hypothesized the C-terminal domain was dispensable for wild type filamentation and growth. To test this hypothesis, we used CRISPR mediated genome editing to generate *ume6-822* which truncated Ume6 after the Zinc-finger domain. We next tested if our mutant strain had a defect in growth rate or filamentation. We found that while *ume6-822* grew at an equivalent rate to wild type at both 30 and 37 C° **Figure 1C,** it had a severe defect in filamentation at both temperatures when grown on Spider media **Figure 1D** . We observed a wrinkled colony phenotype at 37 but not 30 C°, and we never detected agar invasion even after 9 days of incubation at either temperature. This is similar the phenotype observed when we removed the entire Zinc-finger domain and in the deletion mutants (Evans et al. 2018; Zeidler et al. 2009) . Individual cells from the colonies were observed under a microscope, but the heterogeneous nature of these cells makes it difficult to determine if cells in the mutant colonies have distinct morphology from wild type. In liquid spider media *ume6-822* filamented at wild type levels suggesting a specific defect in agar invasion, but not filament formation **Figure 1E** .

Next, we wanted to determine if particular C-terminal tail residues were important for function. We used CRISPR to generate 3 strains; *ume6-833* , *ume6-838* , and *ume6-840,* each of which replaced two C-terminal tail residues with alanine **Figure 1 A and B** . We found these mutants did not change *C. albicans* growth rate and did not have profound effects on filamentation. *ume6-840* appeared to have a minor effect on filamentation, but we are hesitant to place high significance on this change as it was not nearly as severe as *ume6-822.* Attempts to create further truncations and additional mutants were unsuccessful suggesting this region of DNA is not readily accessible to genome editing machinery. Another formal possibility is that these residues are essential in the context of the rest of the protein. Ume6 is not essential, as much more severe truncation and deletion mutant strains are viable (Evans et al. 2018; Zeidler et al. 2009) .

While the ramifications of *UME6* deletion on *C. albicans* biology have been thoroughly characterized, little is known about how the underlying biochemistry of the Ume6 protein leads to these phenotypes. To further understand why the truncation of the poorly conserved Ume6 C-terminal tail had such a profound effect on filamentation, we examined structural predictions for Ume6. Alphafold structural predictions are provided on the *Candida* genome database (CGD) for all predicted open reading frames in the annotated genomes (Skrzypek et al. 2017) . The Alphafold predication for *C. albicans* Ume6 provided little insight into the function of the N-terminal domain as almost the entire domain had confidence scores below 50 which is indicative of a protein region that either lacks structure or is labile (Jumper et al. 2021) . This lack of confidence was conserved in the Alphafold predictions for the *C. dubliniensis* , *C. parapsilosis* , and *C. auris* Ume6 homologs. The Zinc-finger and C- terminal domains however are predicted with high confidence with the Zinc-finger forming a classic Zinc-finger fold and the C-terminal domain predicted to form a single alpha helix. Furthermore, Asn825’s side chain amine is predicted to hydrogen bond to the carboxylic acid backbone of residues Phe791 and Gly792 located in the Zinc-finger domain. Analogous interactions are predicted in *C. dubliniensis* and *C. parapsilosis* Ume6 homologs. *C. auris* , the only other Ume6 homolog for which Alphafolds predictions are given on the CGD is predicted to form a C-terminal alpha helix, but the residues that the *C. auris* helix uses to interact with the Zinc-finger domain are distinct from the predictions for *C. albicans* , *C. dubliniensis* and *C. parapsilosis* .

Taken together our data suggests an important conserved role for the Ume6 C-terminal tail in the CTG fungal clade. In *C. albicans* , elimination of the C-terminal tail leads defects in filamentation. While none of the point mutants we made led to an observable defect in filamentation, Alphafold predictions suggest hydrogen bonds between the Asn825 side chain and the Zinc-finger motif could be important for orienting the Zinc-finger motif. In addition, Zn-finger proteins have been shown to dimerize and the C-terminal tail interactions could play a role in orienting the Zinc-finger domain so that dimerization can take place (Laity et. al 2001) . Phe791 and Gly792 are highly conserved and are two of the residues that comprise the turn in between the two sets of two cysteines predicted to coordinate the Zn ^2+^ ion. The interaction between Asn825 and both Phe791 and Gly792 or an analogous interaction is predicted to occur in a number of other CTG species suggesting a shared structural theme of a C-terminal alpha helix that interacts with the Zinc-finger domain via hydrogen bonds. Interestingly, *C. glabrata’s* Ume6 C-terminal tail is predicted to form an alpha helix but no hydrogen bonds are predicted to interact with the Zinc-finger domain. Alphafold was not able to predict the structure of the vast majority of *C. albicans* Ume6, suggesting these regions are either unstructured or highly labile. To our knowledge, there are no known structures of the Ume6 protein family in the protein data bank, but many budding yeast species encode Ume6 homologs. The effect of Ume6 on phenotype differs between species, with the *S. cerevisiae* homolog thought to be important for mating and *C. albicans* essential for filamentation. Furthermore, while Ume6 has been shown to play a role in filamentation in a number of CTG clade species, the process and significance of filamentation to virulence varies amongst members of the CTG clade.

## Methods

*C. albicans* Strain Creation:

Guide RNA primers targeting *UME6* were cloned into pV1093. Vectors were linearized via digestion with KpnI and SacI. Repair templates encoding premature stop codons or point mutations in *UME6* were synthesized. Repair templates and linearized vector were co-transformed into *C. albicans* SC5314 via lithium acetate transformation. Transformations were plated on YPD supplemented with 200 µg/ml nourseothricin for 3 days at 30°C. Single colonies were isolated on YPD supplemented with 100 µg/ml nourseothricin and grown for 3 days at 30°C. Mutants were identified via restriction digest screening and DNA sequencing.

Growth and Filamentation Assays:

Strains were grown overnight in YPD at 25°C. Each strain was diluted to 0.1 OD _600_ . 4-fold serial dilutions were plated on YPD and Spider. Plates were incubated at 30 and 37°C. Images were taken from 24 hours to 9 days. For liquid filamentation strains were grown in spider media for 24 hours at 37 °C and visualized using a Leica DMIL microscope.
